# Household

**Published:** 1887-04

**Authors:** 


### HOUSEHOLD.

How to Make Coffee —It is not good policy to purchase coffee ready ground, but if it must be done the supplies should be small and frequent Any one may test the purity of ground coffee by shaking a little over a tumbler of clear, bright, cold water, and leaving it for an hour or so. Pure coffee communicates its color to water slowly, and when the color has been imparted the infusion is still bright and clear, and the color is never deep. But chicory and other adulterants quickly produce an opaque and dark infusion. The difference is so striking that for ordinary purpose a better test is not required. To place good coffee on the table daily is a simple and inexpensive business, but it cannot be done at a penny a cup, as some folks are in haste to aver. At for 12 to 20d. per pound a good coffee in berry is always obtainable, and 16 pence may at the present time be considered a fair family price. It is best to roast and grind as wanted, but the grinding is the one important point, because ground coffee quickly parts with its aroma, and there is a great charm in having it made immediately from the mill. In some houses the trouble of grinding is thought much of, but as a matter of fact, it is almost nothing, and a mill costing only a few shillings will last a lifetime. Coffee should never be boiled ; it should be made with soft water at boiling heat, but if hard water must be used it should not be made to boil until wanted, for boiling augments its hardness. A common tall coffee-pot will make as good coffee as any patented invention, but a *cafetiere* is a convenient thing as it produces bright coffee in a few minutes, and thus enables us to secure a maximum of the aroma and dispense with the use of any rubbish called "finings." Every one to his taste, we will say, but as careless people make the coffee too strong one day and too weak the next, the ground coffee and the boiling water should be both measured, and it will always take as much as four cups of water to make three cups of coffee. For the breakfast table the addition of about one-eighth of chicory is an improvement, but for the dinner table coffee should be made without chicory, because it dulls the piquant flavor of the genuine article. Two points in coffee making deter people from using it—the trouble of grinding and the boiling of the milk. The grinding, however must be done, and it is really nothing, but the boiling of the milk can be advantageously evaded by using Swiss milk, which harmonizes perfectly, and by many well-trained palates is preferred to fresh milk heated.

Home Notes.—"Order is Heavens first Law," and "Cleanliness is that virtue which is next to godliness." Both these traits, it is hardly necessary to suggest, are imperatively requisites in good housekeeping. It is the rightful part of the feminine head of the house to study the home interests, to perfect herself in their management, and if she has not personally to do, to carefully and judiciously superintend all the workings of the domestic machinery, and keep all the wheels well oiled and in motion. She is none the less a true woman, (lady, if I must say it, to be understood,) for being able to do it; able to control with intelligence and an unerring hand all the minutiae of the domestic economy, and equally at home, at ease, in the kitchen and the parlor. Who thinks less of her for this? who does not respect her more! To put her own hands intelligently to the different processes that conspire to the comforts of the family, the pleasures of home if need be, is no possible disgrace. To do it *well*, is a credit, surely. Who considers it any disparagement to say "she can get up a delightful dinner?" Now-a-days ability in this department passes for a solid accomplishment, is considered a "fine art." Order makes some duties light and burdens easy. It saves time, too, so that no moments are lost in searching for mislaid utensils, no thoughts wasted, in "wondering where things *can* be," for they all have a regular place, and are *kept there*, and then readily found when wanted, but with all implements and materials in confusion, at sixes and sevens, nothing can be done satisfactorily, time is wasted, patience exhausted, and all goes wrong; housekeeping soon looses all its charms and pleasures. Order must be habitual with ourselves, if we would exact it in subordinates. We should be a pattern of the virtue, if we would instil it:

Neatness is next in importance to order. No one ever heard of a well kept home where neatness did not "reign supreme." It is a trait that is given by nature in a generous degree to some, and in a most lamentably economical measure to others. It is useless to try to teach those who serve us to be neat and cleanly, if there is not some idea or principle of tidiness *born in them*. But give loose reins to one possessing this invaluable quality and it will be evident everywhere in the house; the edibles will be neatly cooked, and prepared to look inviting, and put on the table, in proper and tasteful order, the kitchen floor will be as white as the "deal tables" of our grandmothers time, the kitchen ware will all have its appointed place, be as bright and shining as silver itself. The various rooms kept well swept and garnished, with none of the rugs lying away, the beds "made up by square rule"—a place for everything and everything in its place.

Only give these two qualifications, order and neatness, their rightful weight and value in the management and execution of domestic matters, and half the care and burdens, and all the terrors of housekeeping will soon disappear.—*The Earth.*

Aunt Kittie's Suet Pudding.—One cup of molasses, one cup suet, one cup raisins, one cup of milk. two teaspoonfuls baking powder: add flour till very stiff to beat with spoon; put in a steaming-pan or floured bag, and steam constantly for three hours.

White Cake.—One cup of butter, three cups of sugar beaten to a cream; four cups of flour and half cnp of corn starch, added alternately with a cup of sweet milk, two teaspoonfuls baking powder, flavor to taste; lastly the whites of twelve eggs beaten to a stiff froth.

Puff Pudding.—One pint of boiling milk and nine tablespoonfuls of flour, mix first with a little cold milk. When cold add a little salt and flour, well-beaten eggs and bake in a buttered dish. Serve at once.

